# Annular elastolytic giant cell granuloma associated with autoimmune hepatitis: Response to ciclosporin

**DOI:** 10.1002/ski2.393

**Published:** 2024-06-03

**Authors:** Zaina Sharif, Shagayegh Javadzadeh, Jeanne Boissiere, Daniel Creamer

**Affiliations:** ^1^ Department of Dermatology King's College Hospital NHS Foundation Trust London UK

## Abstract

A 47 year old Caucasian female with a background of type 2 diabetes mellitus, hypothyroidism, and autoimmune hepatitis presented with a painful, pruritic, papular eruption in a photosensitive distribution across the upper chest, neck, face, dorsal hands and forearms. On examination, lesions coalesced into annular plaques each with an active, raised margin and an atrophic, yellow centre. Histopathology demonstrated an absence of mucin and elastophagocytosis with giant cells engulfing dermal elastin fibres. These histopathological features favoured a diagnosis of annular elastolytic giant cell granuloma (AEGCG). The patient was managed ciclosporin monotherapy 125 mg twice daily (3 mg/kg/day). At 8 week review, there was a marked improvement in the physical appearance of the dermatosis as well as diminishing of symptoms such as itch and cutaneous pain. AEGCG is a rare inflammatory dermatosis typically affecting sun‐exposed sites. It has been proposed that AEGCG is triggered by a solar induced elastolysis however other theories suggest it is a primary granulomatous disorder and not a photodermatosis. AEGCG appears to be aligned to an autoimmune diathesis, indicated by its frequent association with autoimmune conditions such as Hashimoto’s thyroiditis, vitiligo, giant cell arteritis and, as in our patient, auto‐immune hepatitis. Diabetes mellitus occurring concurrently with AEGCG has also been observed, again like our patient. Histopathological features which distinguish AEGCG from granuloma annulare include absent mucin, absent necrobiosis, giant cells with more nuclei, non‐palisading granulomata and marked loss of elastic tissue. AEGCG is often unresponsive to standard therapies. The literature indicates varying responses to photo‐protection, topical/systemic/intralesional corticosteroids, and oral medications such as methotrexate, hydroxychloroquine, and dapsone. Few case reports have also documented improvement with ciclosporin. In aggressive forms of AEGCG, as in our patient, treatment with ciclosporin may be an effective intervention and should be initiated early in the disease.

## Graphical abstract

A 47 year old Caucasian female with a background of type 2 diabetes mellitus, hypothyroidism, and autoimmune hepatitis presented with a painful, pruritic, papular eruption in a photosensitive distribution across the upper chest, neck, face, dorsal hands and forearms. Lesions coalesced into annular plaques with an active, raised margin and an atrophic, yellow centre. Histopathology demonstrated an absence of mucin, lack of necrobiosis and elastophagocytosis, favouring a diagnosis of annular elastolytic giant cell granuloma. Ciclosporin monotherapy demonstrated a marked improvement in the physical appearance of the dermatosis and an improvement in itch and cutaneous pain on 8‐week review.

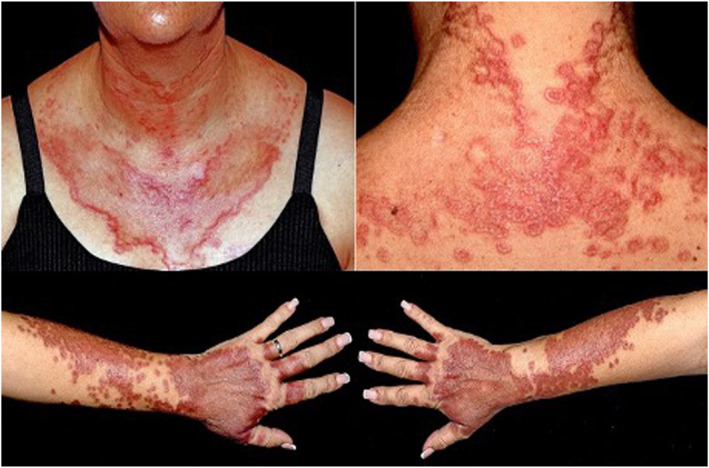

## 1 CASE REPORT

**FIGURE 1:**
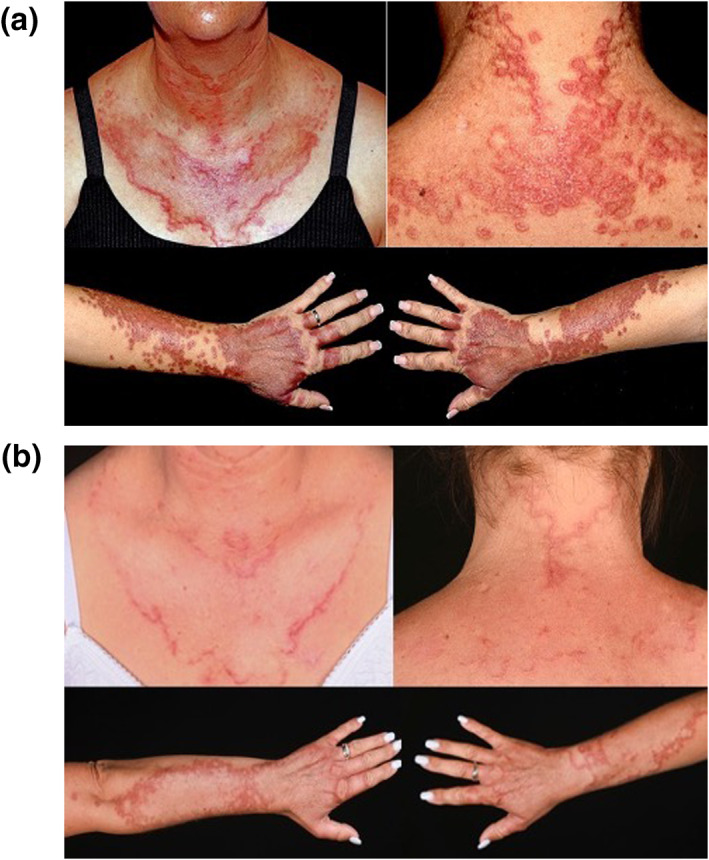
(a) Photo‐distributed rash with an active, erythematous margin, and yellow atrophic central region; (b) resolution of rash following 8 weeks of ciclosporin monotherapy.

A 47‐year‐old Caucasian female with a background of type 2 diabetes mellitus, hypothyroidism, and autoimmune hepatitis presented with a painful, pruritic, papular eruption in a photosensitive distribution across the upper chest, neck, face, dorsal hands and forearms. Lesions coalesced into annular plaques each with an active, raised margin and an atrophic, yellow centre (Figure 1a). The patient did not have a history of significant sun exposure. Routine medications included prednisolone 15 mg once daily, mycophenolate mofetil 500 mg twice daily, empagliflozin, metformin, insulin, and ursodeoxycholic acid. The clinical differential diagnosis for her dermatosis included granuloma annulare, actinic granuloma, necrobiosis lipoidica and annular elastolytic giant cell granuloma (AEGCG).

**FIGURE 2:**
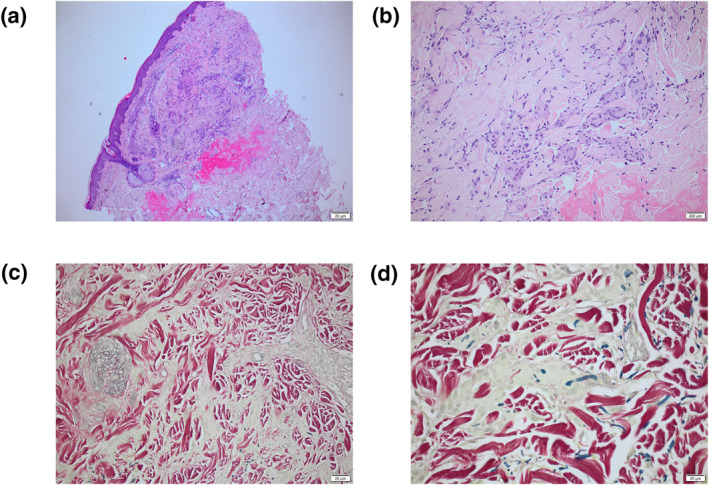
(a) Haematoxylin and Eosin (H&E) stain × 40. Skin biopsy from the edge of the lesion revealing a superficial dermal infiltrate; (b) H&E stain × 200. Skin biopsy from the edge of the lesion revealing a mixed inflammatory cell infiltrate rich in giant cells but lacking necrobiosis and mucin deposition; (c) EVG stain × 200. Skin biopsy from the edge of the lesion demonstrating a paucity of elastin (blue) compared with surrounding collagen (red); (d) EVG stain × 400. Skin biopsy from the edge of the lesion revealing elastophagocytosis. EVG, elastic Van Gieson.

An incisional biopsy was performed sampling the active margin and the adjacent atrophic, central zone of lesional skin. Histopathology demonstrated a superficial dermal histiocytic infiltrate with a predominance of giant cells and an absence of mucin. There was no evidence of necrobiosis. The elastic Van Gieson (EVG) stain demonstrated elastophagocytosis with giant cells engulfing dermal elastin fibres resulting in loss of elastin from the atrophic centre of the lesion but with collagen fibres remaining (Figure 2). These histopathological features favoured a diagnosis of AEGCG.

The patient was treated with super‐potent topical corticosteroid ointment and hydroxychloroquine 400 mg once daily. After 9 weeks of this regimen there had been no improvement in symptoms and an extension of the dermatosis. Treatment was switched to ciclosporin 125 mg twice daily (3 mg/kg/day). At the 8‐week review, ciclosporin monotherapy had induced a marked reduction in the physical appearance of the dermatosis and a significant lessening of both itch and cutaneous pain (Figure 1b).

## 2 DISCUSSION

AEGCG is a rare inflammatory dermatosis typically affecting sun‐exposed sites. A predilection for uncovered skin has led to the hypothesis that UV‐induced damage to dermal elastic fibres triggers a CD4‐mediated T cell response.^1^ Other authors have suggested that AEGCG is a primary granulomatous disorder and not a photodermatosis.^1^ AEGCG appears to be aligned to an autoimmune diathesis, an observation indicated by its frequent association with autoimmune conditions such as Hashimoto's thyroiditis, vitiligo, giant cell arteritis and, as in our patient, auto‐immune hepatitis.^1^ Diabetes mellitus occurring concurrently with AEGCG has also been observed, again like our patient. There is evidence to suggest that diabetes is associated with structural damage of elastin fibres, a typical histological feature of AEGCG.^1^, ^2^

Histologically AEGCG is characterised by a granulocytic, dermal infiltrate with disruption and loss of elastin fibres by elastophagocytosis, findings best demonstrated by biopsying the elevated edge of a plaque.^3^, ^4^ Histopathological features which distinguish AEGCG from granuloma annulare include absent mucin, absent necrobiosis, giant cells with more nuclei, non‐palisading granulomata and marked loss of elastic tissue.^1^

AEGCG is often unresponsive to standard therapies. The literature indicates varying responses to photo‐protection, topical/systemic/intralesional corticosteroids, and oral medications such as methotrexate, hydroxychloroquine, and dapsone.^3^, ^4^, ^5^, ^6^, ^7^ Two case reports have documented improvement with ciclosporin, the first after 8 weeks of 5 mg/kg/day^6^ and the second following 7 months of 200 mg once daily.^4^ Another case report has also described response to ciclosporin and dapsone in combination.^5^ In the absence of effective, active therapy, the literature describes AEGCG as being a self‐limiting disorder, although resolution can take up to 10 years.^7^ The symptom burden in AEGCG varies widely: some patients report mild itch only, others have troubling disfigurement, and few complain of severe pruritus and pain. In aggressive forms of AEGCG, as in our patient, treatment with ciclosporin may be an effective intervention and should be initiated early in the disease.

## CONFLICT OF INTEREST STATEMENT

The authors declare no conflicts of interest.

## AUTHOR CONTRIBUTIONS

**Zaina Sharif**: Formal analysis (lead); writing—original draft (lead); writing—review and editing (lead). **Shagayegh Javadzadeh**: Formal analysis (supporting); writing—original draft (supporting); writing—review and editing (supporting). **Jeanne Boissiere**: Investigation (supporting). **Daniel Creamer**: Supervision (supporting); writing—review and editing (supporting).

## ETHICS STATEMENT

Not applicable.

## Data Availability

Data sharing is not applicable to this article as no new data were created or analysed in this study.
